# Is lap nephrectomy superior to open? A randomized trial

**Published:** 2008

**Authors:** Anup Kumar, Rajeev Kumar

**Affiliations:** Department of Urology, All India Institute of Medical Sciences, Ansari Nagar, New Delhi, India

## SUMMARY

This prospective randomized trial compared laparoscopic simple and radical nephrectomy with open surgery. Between 2001 and 2004, 45 patients requiring nephrectomy for benign and malignant disease (tumors up to 8 cm, confined to kidney) were randomized to either open or laparoscopic group. Open nephrectomy was performed using a 11^th^ or 12^th^ rib flank incision, while laparoscopic nephrectomy was performed using a standard transperitoneal technique. The specimen was removed intact in a sealed retrieval bag, after enlarging ipsilateral iliac fossa port site incision. Various parameters including patient demographics, operating time, mean blood loss, median postoperative hospital stay, mean time to recovery, intra and postoperative complications, pre and postoperative mean visual analog pain scores (at third day, three months and 12 months), quality of life scores (Euro- QOL-5D at three and 12 months) were recorded for the two groups and analyzed statistically. There were 24 patients in the laparoscopy group and 21 patients in the open group. Benign and malignant renal diseases were 19 and five respectively in the laparoscopy group, and 12 and nine respectively in the open group. The two groups were comparable regarding age, sex and weight. The mean operating time (105 min vs. 93 min), mean blood loss (505 vs. 540 mL) and intra and early postoperative complications were comparable in the two groups. There were two open conversions due to bleeding. However, three patients in the open group (two-incisional hernia, one-painful scar) and none in laparoscopy group developed late postoperative complications. The mean visual analog pain scores were significantly lower on postoperative Day 3 in the laparoscopy group (*P*=0.02). However, Euro-QOL scores were comparable in the two groups at three and 12 months. The postoperative recovery was faster in the laparoscopy group as compared to the open group (41 vs. 62 days, *P*=0.04). The authors concluded that laparoscopic nephrectomy results in lesser postoperative pain, fewer late postoperative complications and faster return to normal activities than open nephrectomy.

## COMMENTS

Laparoscopic nephrectomy has become a well-established option for localized renal tumors (T1-2N0M0) with a number of series reporting success equivalent to that of open surgery. Comparisons between laparoscopic and open radical nephrectomy have consistently shown advantages in favor of the laparoscopic approach with regard to all indices of peri-operative morbidity, including estimated blood loss (EBL), postoperative narcotic requirements, length of hospitalization and duration of convalescence.[1–5] However, most of the previously reported series were retrospective or non-randomized studies. This is the first prospective randomized trial comparing open and laparoscopic nephrectomy. The authors have also concluded in favor of laparoscopic nephrectomy regarding lesser postoperative pain, fewer late postopeartive complications and faster return to normal activities, as compared to open nephrectomy.

However, there are several limitations to the present study. The sample size in the two groups is too small to get statistically significant results. The authors have included both benign and malignant renal disease for the nephrectomy. Complications associated with nephrectomy for renal malignancy may be different as compared to nephrectomy for benign renal disease. Parasitic vessels in renal tumors, with increased tumor size add to technical difficulty of laparoscopic nephrectomy, with increased risk of bleeding complications. The authors have not mentioned the reason of preferring transperitoneal approach to retroperitoneal approach, especially for benign renal disease and small localized renal tumors. The mean analgesic requirement in mg morphine equivalent (objective assessment of pain) of the two groups in the postoperative period should have been recorded along with visual analog scale (subjective assessment of pain), for better interpretation of postoperative pain in patients. Despite these shortcomings, the study confirms previous findings in a more rigorous study design.
